# Access to health care for uninsured Latina immigrants in South Carolina

**DOI:** 10.1186/s12913-018-3138-2

**Published:** 2018-05-02

**Authors:** John S. Luque, Grace Soulen, Caroline B. Davila, Kathleen Cartmell

**Affiliations:** 1 Institute of Public Health, Florida A&M University, Science Research Center, 1515 South MLK Blvd. Suite 207B, Tallahassee, FL 32307 USA; 2 Department of Public Health Sciences, Medical University of South Carolina, Charleston, SC USA; 3 College of Nursing, Medical University of South Carolina, Charleston, SC USA

## Abstract

**Background:**

South Carolina is considered a “new destination” state for Latino immigrants. Language barriers, transportation difficulties, low socioeconomic status, inflexible work schedules, different cultural norms, and anxiety and fear related to the current anti-immigrant political climate all negatively impact Latino immigrants’ frequency of contact with the health care system, and consequently they suffer poor health outcomes. The study objective was to explore uninsured Latina immigrant women’s access to health care and alternative treatment strategies in coastal South Carolina.

**Methods:**

The study design was a qualitative interview design. Thirty women participated in semi-structured interviews in community sites. Thematic analysis identified salient categories of topics across interview participants.

**Results:**

The themes were organized into four primary categories including: 1) Barriers and Facilitators to Healthcare, 2) Health Behaviors and Coping Mechanisms, 3) Disease Management Strategies, and 4) Cultural Factors. Participants demonstrated determination for accessing care but reported that their primary health care access barriers included the high cost of services, lack of health insurance, family and work responsibilities, and language barriers. Coping mechanisms included activating their social networks, visiting family and friends and assisting one another with navigating life challenges.

**Conclusion:**

Participants overcame obstacles to obtain healthcare for themselves and their family members despite the multiple barriers presented. Social networks were leveraged to protect against some of the negative effects of financial barriers to health care access.

**Electronic supplementary material:**

The online version of this article (10.1186/s12913-018-3138-2) contains supplementary material, which is available to authorized users.

## Background

South Carolina is considered a “new destinations” state for Hispanics/Latinos, like Georgia and North Carolina [1]. The Hispanic/Latino population in South Carolina grew by 148% between 2000 and 2010 and currently represents around 5% of the population of the state [2]. Compared to non-Hispanic Whites, Hispanics/Latinos, hereafter referred to as Latino/a, are less likely to have health insurance, and more likely to have lower educational attainment and lower median household incomes [3]. For Latino immigrants specifically, health and income disparities are even more pronounced, and the current US immigration policy, which essentially criminalizes unauthorized immigrants, is associated with negative health outcomes for all Latinos, irrespective of immigration status [4–6]. According to a conceptual model developed in a community-engaged study in Massachusetts, the anti-immigrant climate caused immigrants to experience fear and distrust of institutions, which in turn led to increased feelings of marginalization, poor mental health, and missed appointments or unfilled prescriptions [7]. These negative health behaviors and practices ultimately led to negative health outcomes such as uncontrolled hypertension or diabetes and were also associated with a lack of preventive health screenings.

Previous studies have reported that recent Latino immigrants are less likely to be knowledgeable about access to health care and other health-related resources (e.g., food assistance) than Latino immigrants who have lived in the US for a longer period [8, 9]. While the Patient Protection and Affordable Care Act (ACA) of 2010 extended health insurance coverage to many uninsured individuals, undocumented immigrants were specifically excluded from purchasing health insurance coverage through the health exchanges created by the law [10, 11]. Language barriers, transportation difficulties, low socioeconomic status, inflexible work schedules, different cultural norms in the US, and anxiety and fear related to the anti-immigrant political climate negatively impact Latino immigrants’ frequency of contact with the health care system [7, 12–16].

The purpose of this qualitative study was to explore Latina immigrant women’s access to health care for both preventive screenings and medical care in the coastal region of South Carolina. Our two primary research questions were: (1) What are the major individual and structural barriers facing this population for accessing health care? and (2) What are some of the alternative health care seeking strategies used by Latina immigrant women and corresponding cultural beliefs surrounding these practices? This study examined sociocultural factors associated with preventive health care for common chronic diseases affecting Latino immigrant women, such as cancer, cardiovascular disease, diabetes, and hypertension. The study also employed a community-engaged strategy to identify and address the most salient health issues from the community’s perspective.

## Methods

Thirty Latina immigrant women participated in a semi-structured qualitative interview facilitated by the third author, a native Spanish-speaking woman, in fall/winter 2016. Interview questions included standard close-ended survey questions for demographic and health history data and open-ended qualitative questions adapted from a similar study of health care access for an immigrant community in North Carolina (refer to Additional file 1) [17]. We employed purposive sampling to recruit uninsured, Latina immigrant women between the ages of 21 to 64 years old who lived in the tri-county Charleston metropolitan area. Interview sites included two free health clinics, two churches, a suburban flea market, and a community center. Fourteen participants were recruited from the free health clinics, six participants from churches, and 10 participants from the community. Audio-recorded interviews were conducted in Spanish with study participants following verbal informed consent. The study was approved by the Institutional Review Board at the Medical University of South Carolina. Interviews ranged in length from 45 min to an hour. Participants received a $20 stipend and a list of community resources. The data transcription and analysis were all completed in Spanish. The data were analyzed with MAXQDA software using thematic analysis and the constant comparative method [18]. We developed a codebook and detailed code definitions with accompanying example quotes for each code (see Table 1), and interrater reliability was established between the second and third author based on percent agreement using a small sample of half of the interviews until percent agreement reached over 90%. Interviews were transcribed in Spanish in batches by a third-party company. Next, the two coders proceeded to code all the Spanish transcripts until all the interviews were completed. A third coder, the first author, then resolved any disagreements in the coding. Summaries were created for each code category with example quotes listed under corresponding themes which emerged from the coding activities.

**Table 1:** Codebook with code definitions and typical exemplars

Theme	Code	Definition	Quote
Barriers and Facilitators to Health Care	Barriers	Use for any reference to barriers such as lack of insurance, transportation, documentation, language, lack of access to health care	“The first [barrier] is the language, after that it’s not having health insurance and then not having money to pay for the appointments.” - Participant #4
	Community Positives	Apply when individuals mention a positive experience with their community such as health care settings, at work, or in stores	“As Latinos… we receive a lot of good information from the church and the children’s school, they give us good resources about where we can go if we need this or that.” - Participant #15
	Community Negatives	Apply when individuals mention a negative experience with their community such as health care settings, at work, or in stores	“One time I felt discrimination… [a nurse at Northwoods] said that Latinos are very hot-headed, and that we’re only here to cause a burden on the country. I don’t know if it was just a bad moment for her, and she was taking it out on me, but it made me feel bad.” - Participant #28
	Cost of Healthcare	Use for any reference to cost of health care services or lack of health insurance	“I think I would have to be dying before I put myself in more debt and burden myself with a $1000 bill from an emergency room.” - Participant #1
Health Behaviors and Coping Mechanisms	Positive Health Behaviors	Use for any reference to healthy behaviors or recommendations from doctors or family members	“If you’re trying to eat healthy, the healthiest things come from the Earth; vegetables and all that. So, I think herbal remedies and teas are good because they come from the Earth.” - Participant #25
	Negative Health Behaviors	Use for any reference to unhealthy behaviors or recommendations from doctors or family members	“We used to eat healthy back in Guatemala. Everything was natural there and there wasn’t as much disease. But here, everything is frozen, the nutrition is very different.” - Participant #13
	Coping Mechanisms	Use for any reference to how participants cope with anxiety or depression including their support systems	“We try to avoid depression and feel positive but sometimes it’s hard because we don’t speak much English, so we gravitate to other immigrants. I feel good when I can help other women with whatever I can. I’m also Catholic and we have support groups that give us strength… we pray together and share our experiences to keep our minds healthy.” - Participant #24
Disease Management	Clinic Choice	Use for any reference to health care settings, including free or low-cost clinics	“It’s very expensive to go to the emergency room. So, for routing health you go to different places where they give you free or low-cost care.” - Participant #23
	Disease Management	Use for any reference to challenges and/or successes with disease management, mainly chronic diseases such as diabetes or hypertension	“Well if something hurts… you wait until you feel really sick and then you run to the doctor. In the meantime, you take a pill or something for the pain.” - Participant #26
	Disease Perceptions	Use for any reference to conditions or diseases that affect the Latino community in Charleston and in the US	“I don’t think anyone has control [of their own health], but we do have lots of control over our nutrition and taking care of ourselves.” - Participant #8
	Medications	Use for any reference to over the counter or prescription medications	“We go to the Mexican stores to buy our pills, Tylenol, all of it.” - Participant #1
	Signs of Illness	Use for any reference to signs or symptoms that cause someone to seek healthcare	“The body tells us when we’re sick, but sometimes we don’t listen.” - Participant #15
Cultural Factors	Role of Alternative Medicine	Use for any reference to the use of herbal teas, alternative medicine, massages and where they obtain the products or services	“My mom would say to drink a lot of tea, like chamomile tea, also garlic on an empty stomach … it helps with a lot of things.” - Participant #26
	Role of Family	Use for any reference to how family impacts their decisions and lives	“When my daughter was little I would go with my husband, and he understands English.” - Participant #17
	Role of Religion	Use for any reference to how religion or being involved in church activities impacts their decisions and lives	“Yes, I look for Christ because I think that it helps; Professional therapy also helps but I think of looking to God first.” - Participant #21

In the following section, we report the qualitative findings and describe the major themes that emerged from the data. Illustrative quotes were translated from Spanish to English. Different participants are listed by ID numbers with basic demographic characteristics (age, marital status, and country of origin) to protect confidentiality and differentiate interview participants.

## Results

### Sociodemographic characteristics

Table 2 details the sociodemographic characteristics and health status of the 30 study participants. Participants had an average age of 40, ranging from 26 to 63 years old. Most women were married, unemployed, and from Mexico. The average length of time they had been living in the US was 14 years, ranging from 3 to 27 years. Participants also had limited formal education, low household incomes and did not speak English “very well or well.” None of the participants had health insurance at the time of the interview, but four participants reported they had sought health insurance coverage in the last three years (since the implementation of the ACA), and 10 participants did not have a regular medical provider. Many participants reported having a chronic health condition such as diabetes or hypertension, and 7 participants reported mental health issues, such as depression or anxiety. Regarding preventive screenings, 26 participants were up-to-date with cervical cancer screenings, three out of 12 women aged 40 years and older were up-to-date with screening mammograms, and 21 women had received their last blood glucose test within the past 12 months. Finally, 14 women had received their last routine check-up within the past 12 months.

**Table 2:** Sociodemographic characteristics and health status of Latina immigrant women

Characteristics	Total (*n* = 30)	%^c^
Age group		
21–30 years	3	10
31–45 years	19	63
46–64 years	8	27
Marital status		
Single/Separated	4	13
Married	20	67
Domestic partner	6	20
Education		
< 11 years	21	70
12 years or HS	6	20
Some college, tech, or higher	3	10
Employed		
Yes	6	20
No	24	80
Annual household income		
$0 to $14,999	4	13
$15,000 to $19,999	12	40
$20,000 to $34,999	13	43
$35,000 to $49,999	1	4
Speak English		
Very well or well	6	20
Not well or not at all	24	80
Country of origin		
Mexico	24	80
El Salvador	2	7
Guatemala	2	7
Honduras	2	7
Time living in US		
< 10 years	4	13
≥ 10 years	26	77
Number of children < 18 years in household		
None	6	20
1 child	7	23
2–3 children	17	57
Sought health insurance coverage since October, 2013		
Yes	4	13
No	26	77
Have a regular provider		
Yes	20	67
No	10	33
Last routine check-up		
Within past year	14	47
Within past 2 years	8	26
Within past 5 years	5	17
5 or more years ago	3	10
Number of times going to doctor in last year, not including emergency room		
Never	5	17
1 time	7	23
2 times	5	17
3–4 times	10	33
5–9 times	3	10
History of smoking cigarettes		
Yes	3	10
No	27	90
Self-reported health status		
Excellent/very good	5	17
Good	13	43
Fair/poor	12	40
Diagnosed Health Conditions		
Diabetes	9	30
High blood pressure	11	37
Heart conditions	2	7
Chronic lung disease	3	10
Arthritis or rheumatism	1	3
Diagnosed chronic health condition^a^		
Yes	20	67
No	10	33
Depression or anxiety disorder		
Yes	7	23
No	22	77
Family history of cancer		
Yes	9	30
No	21	70
Confidence in taking good care of own health		
Completely/very confident	19	63
Somewhat confident	5	17
Little/not confident	6	20
Last Pap test		
A year ago or less	16	53
1–3 years ago	10	33
More than 3 years ago	4	14
Last blood glucose test		
A year ago or less	21	70
More than a year ago	9	30
Last Mammogram^b^		
Within past 2 years	3	25
Over 2 years ago	4	33
Never	5	42

^a^Derived variable from 5 different types of chronic conditions including: diabetes, high blood pressure, heart conditions, chronic lung disease (or asthma, emphysema, chronic bronchitis), and arthritis or rheumatism

^b^Only reported for women aged 40 and over (*n* = 12)

^c^Some overall percentages total over 100% because of rounding

### Thematic analysis

The following sections present the results of the qualitative data analysis. The themes were organized within four primary categories including: 1) Barriers and Facilitators to Health Care, 2) Health Behaviors and Coping Mechanisms, 3) Disease Management Strategies, and 4) Cultural Factors. Within these overarching themes were nested multiple codes including: Barriers, Community Positives and Negatives, Cost of Health Care, Positive and Negative Health Behaviors, Coping Mechanisms, Clinic Choice, Disease Management, Disease Perception, Medications, Signs of Illness, Role of Alternative Medicine, Role of Family, and Role of Religion. The interviews generated discussions related to uninsured, Latina immigrant women’s perceptions of access to health care and treatment or management strategies for various health conditions.

#### Barriers and Facilitators to Health Care

The Barriers and Facilitators to Health Care theme contained the following codes: Barriers, Community Positives and Negatives, and Cost of Health Care. While the study participants described different strategies for accessing care, the primary barriers included the high cost of services, especially for emergency room care, lack of health insurance, family and work responsibilities, and language barriers. These were all more pressing concerns affecting access than other potential barriers mentioned such as transportation or discrimination in health care encounters. Facilitators to health care included access to interpretation services, social support from friends and faith leaders, and availability of low-cost prescriptions. Regarding financial barriers, one participant stated, “One can afford to pay for the doctor’s office visit to know what is wrong with you, but not for the treatment.” (ID #1, age 35–39, Honduras).

Echoing these concerns, another woman explained that she could get an appointment for an eye exam but could not complete the surgery unless she paid $2600. She was unable to arrange for the provider to set up a payment plan and commented, “And so, they were going to make an appointment for my surgery, but I lost the appointment because I could not afford it. So, I told them I could not go, So, they just told me, ‘When you are ready.’” (ID #30, age 40–44, Mexico).

Another participant explained that they once had to pay a very high price for insulin at a pharmacy. However, this participant subsequently found insulin at a lower price at a Wal-Mart pharmacy for between $20 and $40 dollars and explained, “I had a bad experience the first time I went to fill my prescription for insulin. I went to Walgreens and just one vial was $163.” (ID #28, age 45–49, Mexico).

Additional complaints about perceived high costs of prescription drugs and health care services were frequent, particularly since participants did not have health insurance to offset the costs. For instance, some participants lamented the cost of health care and explained that they could not afford it because costs were not offset by health insurance. One participant stated, “It’s very expensive … just one doctor’s visit was $135 and then additionally I had to pay for the medicine. Basically, there went half of my weekly income.” (ID #24, age 40–44, Mexico). Another participant responded, “The big concern with our community is that doctor’s visits are very expensive. One doesn’t have the freedom to say, ‘I’ll just go’ because it’s a lot of money, and we don’t have health insurance.” (ID #2, age 50–54, El Salvador).

However, some participants did report that costs could be reduced at some clinics after submitting some paperwork to justify sliding scale fees. For example, one participant explained, “I had to fill an application, and then take it to have it approved, and then it was signed … I was originally being charged $400 and then I ended up with a $130 charge.” (ID #27, age 25–29, Mexico).

Nevertheless, the lack of insurance and high costs of health care could result in a failure to access care for those with serious illnesses or even lead them to return to their countries of origin to seek care. This sentiment was expressed by one participant.

Personally, what worries me is that if someone gets a serious illness, and because of lack of insurance or money it cannot be treated here. I have seen many people with children that were born abroad and since they don’t have medical insurance, if they happen to get cancer or leukemia they must go back to their country to die because, if there are no resources here, there are even less back home. (ID #4, age 30–34, Mexico).

Another participant expressed a similar attitude and replied, “Well, unfortunately we are seeking help because our earnings are so low. And if one gets sick one doesn’t want to be a burden on the government like they say, so we look for free care, and sometimes one needs to pay to get it [health care].” (ID #24, age 40–44, Mexico).

In addition, because of high medication prices, some patients do not refill their medications, leading to unfortunate outcomes from chronic conditions, such as uncontrolled hypertension. In some cases, patients had to set up payment plans due to their lack of prescription drug coverage. One participant explained, “It’s expensive…it’s a lot of money so sometimes one doesn’t buy the medicine one needs because it’s too expensive. If we get the generic it’s cheaper…In some supermarkets, some prescriptions are free.” (ID #2, age 50–54, El Salvador). Another participant stressed the importance of payment plans by stating, “Well that medicine is costly, but I had to find a way to get it even though the cost was excessive…I had to set up a monthly payment plan.” (ID #3, age 35–39, Mexico).

In addition to costs of seeking a doctor and affording one’s medication, several participants discussed work/life balance issues and the lack of social support as barriers to care. One participant stated, “I am taking care of my children and I don’t have anyone to help me here [in this country].” (ID #7, age 30–34, Mexico). Another participant responded, “Sometimes, well, most of the time we are at work. I go to work and dedicate my time to my job, so my health is affected.” (ID #9, age 50–54, Mexico).

There was a complicated relationship between work and health. One woman explained, “they don’t eat healthy, but they work a lot.” (ID #25, age 25–29, Mexico), indicating that many Latinos spend a considerable amount of time working, with little time to take care of themselves or exercise. Women reported work as both a motivating factor for staying healthy and as a cause of poor health, “We’re so involved with our jobs and we work, work, work, and leave our health for last.” (ID #15, age 35–39, Mexico).

An additional barrier to accessing care was language difficulty. This affected women’s confidence to make medical appointments and understand all the information conveyed during a typical visit. Even though some participants had tried to learn English, they ultimately were not successful. One participant explained, “The issue of language…and many people say, ‘You have been here for so many years, why don’t you learn [English]?’ Well, there are those who have that capacity to learn and then there are those that cannot speak another language.” (ID #9, age 50–54, Mexico). Another participant expressed frustration with the language barrier by stating, “For instance communication, their Spanish and our English [needs to improve] so that we can understand each other; sometimes the language barrier results in nothing getting done.” (ID #13, age 30–34, Guatemala). Another participant responded, “Yes, it is a big, big barrier because I do not speak English well.” (ID #8, age 40–44, Mexico).

Despite it not being a major barrier to care, access to interpreters or information provided in Spanish and lack of transportation was a source of stress for participants, as one participant stated, “I think that because there are less Latino people in this state … in other states with so many Latinos that are accustomed to see this, and here where they battle with people if they don’t speak English well, people get stressed out, and I have noticed this.” (ID #7, age 30–34, Mexico). Another participant stressed the importance of having interpreters available by stating, “If they had just told me: ‘just a minute let me check’ but I could see that they were just passing my piece of paper back and forth and no one told me what was going on … I felt very sad because I needed (interpreter) assistance and no one could help.” (ID #9, age 50–54, Mexico). Regarding transportation, one participant identified the barrier by explaining, “Well, that is an obstacle here, there is no public transportation.” (ID #17, age 60–64, Mexico).

On the other hand, in terms of facilitators to health care, some participants reported that they had not felt discrimination in health care facilities and received interpretation assistance and prescription drug discounts. One participant stated, “Well, yes they gave me an interpreter” (ID #29, age 55–59, Mexico). Another explained, “Well, you can get free medical visits. That helps a lot, the Latino communities … there are times in the community, if they have the medicine, they will give it to you in the pharmacy for free.” (ID #2, age 50–54, El Salvador).

#### Health Behaviors and Coping Mechanisms

The Health Behaviors and Coping Mechanisms theme contained the following codes: Positive Health Behaviors, Negative Health Behaviors, and Coping Mechanisms. The participants’ health behaviors were heavily influenced by the barriers and facilitators operating in their daily lives. The most common negative health behavior reported was unhealthy dietary habits. This negative behavior was discussed as being a consequence of the cultural transition from their native countries in Latin America to the US, where food was natural and not processed or frozen and served as a protective effect against disease.

A general theme emerged that these women had greater access to inexpensive fresh fruits and vegetables in their native countries compared to the US. As a result, Latina immigrants experienced dietary changes and limitations that contributed to negative health behaviors. Additionally, participants acknowledged an unhealthy excess of fat consumption. For example, regarding dietary preferences, one participant replied, “We Latinos eat tortillas, a lot of tortillas, a lot of bread and a lot of grease.” (ID #17, age 60–64, Mexico). Another participant answered, “I had a lot of bloating, because of so much flour, bread, and tortilla.” (ID #26, age 35–39, Mexico). Another participant explained a generational shift in eating habits that were linked with overall health.

[Regarding parents and grandparents] They didn’t get sick like we do today. No, they never ate grease, on the contrary, they ate food without grease, and they never said, my foot hurts, my back, like that, and they weren’t fat, because they were exercising all the time, one hour to walk to the plaza [outdoor market], all the time. (ID #6, age 40–44, Mexico).

Participants were generally able to identify desired positive health behaviors such as drinking lots of water, eating a healthy diet, and getting enough physical activity. During the interview process, many women also identified several preventive measures they would take to stay healthy including blood pressure screening, Pap tests, mammograms, taking vitamins, and getting eight hours of sleep on average. For example, one woman responded, “I believe in a daily thirty-minute walk, going to the gym or whatever it may be, the key is to keep moving.” (ID #5, age 40–44, Mexico).

Strategies for managing depression and anxiety included coping mechanisms for self-help as well as helping others. None of the women sought professional psychological care or medication and relied instead on activities such as listening to music, knitting, reading, exercising, and cleaning to distract themselves from negative thoughts. For example, one participant stated, “The rest of my family is back in Mexico, so I try to listen to music or read, because I really like to read.” (ID #28, age 45–49, Mexico). Some women attributed their anxiety or depression to social isolation experienced from living in a non-Latino community due to language barriers and separation from family in their home countries. In addition, there was fear related to crime and immigration enforcement, as well as consternation when people exhibited racist attitudes. Since the interviews occurred during the 2016 Presidential election season, participants were very sensitive to the anti-immigrant rhetoric on display in the Republican campaign. One participant stated, “Well, I feel worried … how things are going here. They are talking about how Trump is going to remove all the Latinos, and he will get them all out.” (ID #28, age 45–49, Mexico). Another participant expressed fear by stating, “When my girls come home the older one scolds me: ‘Mom, why are all the blinds closed?’ I answer, ‘I like everything closed because it makes me feel better (safer)!’” (ID #30, age 40–44, Mexico). Another participant observed overt discrimination in public places by explaining one instance, “They answer rudely in the stores, and they answer in a loud voice so that everyone around can’t help but hear the answer. This bothered me.” (ID #1, age 35–39, Honduras).

Women also helped each other cope by activating their social networks, visiting family and friends, and assisting one another with navigating life in the US. Many women identified their faith community as a source of social support. Attending church services and joining women’s groups were also identified as coping mechanisms for improving mental health. One participant replied, “We only speak a little English, so we gravitate to other new immigrants. It makes me feel good when I can help other women with whatever I can.” (ID #28, age 45–49, Mexico).

The support they received in their church from fellow church members helped to improve women’s mental health and served as a safety net. One participant explained, “I go on Sundays and we learn in a group, we gather once a month, we are all women. And we pray, we share. That helps a lot with our mental health.” (ID #24, age 40–44, Mexico). Another participant stressed the importance of social support by stating, “I have pastors and brothers of my church to help me.” (ID #21, age 30–34, Honduras).

#### Disease Management Strategies

The Disease Management Strategies theme contained the following codes: Clinic Choice, Disease Management, Disease Perception, Medications, and Signs of Illness. Study participants identified 15 different clinic choices in the Charleston area, the most common being an uptown free clinic, health department clinics, the university hospital and hospital emergency rooms. The uptown free clinic was listed most often because 13 of the 30 interviews were conducted there. A common theme that emerged was the idea of “shopping around” from clinic to clinic based on cost, language access and available services to avoid the high cost of emergency room care.

Some participants reported no clinic preference and could not remember when they last saw a doctor. Participants were wary of incurring debt from an emergency room visit and used it as a last resort. Participants also reported discrimination in clinic waiting rooms due to language barriers and their uninsured status. When asked about dental care, most women had not found an affordable solution, so they avoided going to the dentist in the US. One participant stated, “When I travel to Mexico that’s when I get dental cleanings and checkups.” (ID #24, age 40–44, Mexico). Some local clinics offer free screenings but not free treatments or preventive care. Other clinics only offer free emergency tooth extractions.

Women reported motivating factors for managing their health as well as their thought processes for deciding how to manage their health. A common motivator for these women was their families. They wanted to stay healthy, so they would be able to continue taking care of their children. Another motivating factor for these women was to prevent the progression of chronic diseases, such as high blood pressure and diabetes, which were the most prevalent chronic conditions among participants. As one woman explained, “The doctor told me to exercise, for my blood pressure.” (ID #30, age 40–44, Mexico). Another offered a comment, “I used to be overweight, but since then I decided to walk and walk.” (ID #27, age 25–29, Mexico).

Many women described similar disease management processes for deciding when and how to visit a doctor for their health concerns. At the first sign of illness, they typically consult friends or family members, and many participants explained that they would try alternative medicine first to feel better before visiting a doctor. One participant explained, “Well the first thing we do is ask our aunt or grandparent, ‘these are my symptoms, what could it be?’ before going to the doctor. Then they tell us a remedy and we try it, and if that doesn’t work then we go to the doctor.” (ID #25, age 25–29, Mexico).

Most women reported having a threshold for when they decide to visit a doctor. One participant stated, “If my knee hurts, I sit down for a while and keep doing my work. But if I have a fever or vomiting… that’s when I go to the emergency room.” (ID #24, age 40–44, Mexico). Another participant described her treatment strategy, “Well if something hurts… you wait until you feel really sick and then you run to the doctor. In the meantime, you take a pill or something.” (ID #26, age 35–39, Mexico).

When asked where they get their medications, 14 women reported using Wal-Mart as a primary pharmacy choice for over-the-counter medications. One participant from El Salvador reported difficulties obtaining a prescription from a large chain drugstore by showing a passport for identification. Six women referenced *tiendas mexicanas* (Mexican stores) as a source of over-the-counter medicines, and one participant referenced a former bad habit of buying antibiotics at one of these stores and not taking the medicine correctly. A general theme from these interviews is the idea of self-medication in which women attempt to self-treat their health problems before going to a doctor for a prescription. These women also “shop around” at different pharmacies that offer certain medicines for free, such as free blood pressure medicines at one grocery store chain. Finally, women often choose generic or off-brand medications instead of name-brands as a cheaper option. One participant explained, “In the past we bought [antibiotics] at the Mexican store, but I’ve gotten rid of that bad habit because it didn’t always help.” (ID #15, age 35–39, Mexico). Other participants mentioned shopping at the Mexican grocery stores for their over-the-counter medications. Finally, another woman implied that if the generic was not available, the brand-name prescription medicines were too expensive, as one participant stated, “Sometimes I don’t buy the medicine, because it’s very expensive. But if you go for the generic, it’s cheaper.” (ID #2, age 50–54, El Salvador).

Responses around signs and symptoms of illness served as indicators and cues to action that would cause women to acknowledge their health condition and seek care or treatment. In general, women reported that symptoms are signs of illness from their bodies. One participant explained, “You know your body and you know when something is not right.” (ID #28, age 45–49, from Mexico).

The top three most important community health concerns reported by study participants were: 1) diabetes, 2) obesity, and 3) cancer. When ranking these health concerns, women recalled family history and referenced people they knew in the community with certain diseases. Participants also discussed perceived causes of disease, such as poor nutrition, drinking soda and lack of exercise. One participant explained, “Many people drink a lot of soda, too much soda. They’ll get diabetes and keep drinking soda.” (ID #2, age 50–54, El Salvador).

One participant mentioned that she did not want to take ownership or identify with her disease. She commented, “I don’t like to say my diabetes. The diabetes, it’s not mine, I don’t want to make it mine.” (ID #12, age 35–39, Mexico).

There were mixed responses for women’s perceived control over their own health. Some women believed they had control over their health, while others expressed less control. For example, one participant stated, “Yes, I think I do have [control]. Sometimes I don’t act on it, but I know that I can, I’m just careless sometimes.” (ID #23, age 35–39, Mexico). Another stated, “I don’t have much [control] because I’m not doing everything that I need to do.” (ID #6, age 40–44, Mexico).

#### Cultural Factors

The Cultural Factors theme contained the following codes: Role of Alternative Medicine, Role of Family, and Role of Religion. The role of the family was discussed by most interview participants. Women admitted that when they were sick, they were very depressed because they could not help with housework or attend to their children, expressing traditional gender norms. For example, one participant stated, “I’m like the motor of my family. If I’m sick my home doesn’t function, I don’t work, I don’t cook, I don’t clean I just lay down. This makes you feel bad.” (ID #24, age 40–44, Mexico).

While participants valued the advice of their elder relatives for treatment advice, many women were very dependent on their children for English assistance. Since participants did not speak English very well, they often relied on their children to translate for them in many situations. One participant explained, “Yes, thanks to God, my children have been teaching me to speak English… I want to feel better and be happy with my children. And, the children, they go with you and translate for you… they help you.” (ID #19, age 35–39, Guatemala). Another participant also expressed a similar sentiment by stating, “[My daughters] translate for me, and sometimes, when I need to go to the doctor, I say: ‘When you get home from school, we’re going to the doctor’ [they say] ‘Okay’ and they translate for me.” (ID #30, age 40–44, Mexico).

Most participants spoke about the role of religion in their life and the importance of prayer. One participant stated, “Always everything that I need and when I feel stressed out, what I do is ask her (Virgin of Guadalupe, Patron of Mexico). I pray to her always.” (ID #4, age 30–34, married, Mexico).

Participants felt a sense of community in their churches and a relief from the outside stressors. One participant expressed this sentiment well by stating, “Yes, going to church relaxes me in other words, I love coming to church, to pray for my family, for my sons and daughters, and well for everything…we forget all our problems.” (ID #26, age 35–39, Mexico).

Study participants also employed several home remedies to possibly delay or avoid having to take on the extra out-of-pocket costs that a clinic visit would entail. For example, one participant stated, “Well, my natural remedies. For example, when I’m sick with the flu, my mom always told me to put orange peel and lemon peel to boil with cinnamon. ‘Drink this to get rid of your cold’… yes, yes, various things my mom gave us. ‘If your ear hurts, add oregano.’” (ID #15, age 35–39, Mexico).

Others provided a lot of information about remedies, including specific instructions for recipes. One participant explained,

I make chamomile tea, mint tea, and for anxiety and nerves I make the one of the seven herbs with valerian root…And for the kidneys I use arnica and that plant called horse tail. You boil a quart of water add a pinch of arnica and three threads of that plant. Then boil all that for a few minutes, cool it in a pitcher and drink that water all day and use it for any other cooking that requires water. (ID #4, age 30–34, Mexico).

Some would also opt to use the services of a *sobandera*, or folk healer, who employs massage and natural remedies to heal the sick. One participant explained, “I know several people who have gotten better. My friend suffered with back pain, so she went to see a folk healer and now she is better.” (ID #25, age 25–29, Mexico). Another participant discussed her experience, “I used to go to a folk healer, and she would rub me with some creams like Vaporub.” (ID #20, age 45–49, Mexico).

Finally, participants also expressed the cultural belief in certain folk illnesses, with one participant explaining the case of *mal de ojo*, or ‘evil eye.’ She explained, “When someone is vomiting and having constant diarrhea, this is evil eye. There is a special herb that can be used to cure it to stop the diarrhea and vomiting.” (ID #6, age 40–44, Mexico).

## Discussion

The aims of this qualitative research study were to explore the barriers that Latina immigrant women encounter when attempting to access health care in coastal South Carolina and to identify alternative health care strategies that women pursue, as well as the beliefs underlying these practices. The research inquiry method allowed women to express multiple barriers to health care access through the interview process facilitated by a Latina female interviewer in Spanish, thus allowing for more open dialogue and discussion. Women identified their difficulties in accessing health care because they did not have either the financial resources or health insurance which would help to defray costs, especially in the case of specialty care. Therefore, we concluded from the sum of the interviews that women would oftentimes either delay or forego seeking health care because they perceived that they would not be able to afford the financial costs. Most of the participants lived in low-income households and were being supported by a spouse or domestic partner.

While many immigrant women interviewed had lived in the US over 10 years, they still encountered barriers to health care access. The barrier of health care costs for primary care was addressed by the free and low-cost health care options for the uninsured patient population: free clinics, health departments, and federally qualified health centers offer affordable services that include primary care, vision screening, dental care, vaccinations, STD/HIV testing, pediatric care, and prenatal care. These clinics do not require proof of insurance and accept sliding scale payments based on income. Access to these resources is restricted sometimes by place of residence or household income thresholds. To access the health care women were seeking, immigrant women employed a strategy of “shopping around.” This strategy sometimes involved visiting their regular provider; however, in practice, participants consulted with several providers for different needs. The last resort for uninsured and undocumented patients was the use of emergency Medicaid, which would apply in the case of patient with advanced cancer for example, but we did not encounter these chronically ill patients in this study [19].

Those who are either uninsured or underinsured are effectively ‘priced-out’ of the health insurance market and reliant on emergency room care, where care cannot be refused. The options for the participants in this study were to schedule an appointment with a free or low-cost clinic, seek same-day care at an urgent care center, or seek emergency room services, which was a last resort. Since emergency room care is one of the most expensive forms of health care, the overutilization of these services by uninsured populations such as undocumented immigrants who cannot afford to pay for services is an unnecessary strain on a health system’s resources and is financially unsustainable [20]. While charity care is charged with addressing the health needs of the uninsured, low-income population, their services are limited, and may not meet the needs of this underserved population for specialty care or chronic disease management [21, 22]. This was noted by study participants who complained about the high cost and unaffordability of specialty care services.

In the field of medical anthropology, the term ‘structural vulnerability’ is used to describe how a group of people experience negative health outcomes because of their subaltern position in society, exposing them to economic exploitation and structural violence [23]. Uninsured, Latina immigrants constitute a structurally vulnerable patient population in clinical settings because if they are diagnosed with a serious condition, they frequently do not have access to medical specialists. Therefore, for follow-up care or managing certain chronic conditions, they rely on the coordination and navigation of an overburdened and understaffed charity care network of providers who may or may not have a referral network of uncompensated care options for these patients.

Currently in the US, the inability of some patients to provide proof of citizenship or legal residence combined with a lack of health insurance places them in a structurally vulnerable situation where they face the possibility of being denied health care or receiving substandard care. The Mexican Consulate provides a consular identification card which, participants reported in this study, is diligently renewed every five years and used for identification to receive health care services. The participants shared that they always either carry this card or their passport – for those from Central America. Others shared that they carried identification issued by one of the ‘Sanctuary Cities’ in other states, such as Texas, that have immigrant friendly policies. Despite having these forms of identification, some participants reporting being unable to obtain their prescription drugs from some pharmacies with these ID cards.

Viewing financial and insurance barriers to health care as structural problems which require comprehensive policy and programmatic solutions is one pragmatic approach to address these seemingly intractable problems in the current US health care landscape [24]. As explained in some of the interviews, participants were reluctant to incur substantial debt from medical expenses and would opt to return to their countries of origin for such treatments, which were more affordable, but of lower quality. Moreover, nationalist political rhetoric currently pervading the conservative media seeks to criminalize Latino immigrants, especially from Mexico, who are characterized as ‘undeserving’ of health care and government services and should be deported, even when apprehended for only minor infractions of the law [25, 26].

The stigmatizing of a group of people has ripple effects which negatively affects Latino immigrant’s physical and mental health independent of immigration status [21]. Furthermore, Latino immigrants have a growing mistrust of any institution suspected of sharing information with immigration authorities, and this includes health care facilities [12]. The resource constrained safety net unfortunately fails to catch many it is intended to help, contributing to the narrative that some of society’s members are more deserving of care than others, while the Latino immigrant community remains in a precarious and structurally vulnerable situation in terms of equitable access and may also experience negative health care encounters [27].

Through the course of interviews, a theme emerged that the delaying of accessing health care services was associated with an internalized feeling of not being worthy or deserving of care, as evidenced by not wanting to be perceived as using government assistance programs reserved for US citizens. The indirect result of delaying or avoiding health care had several negative consequences which included worsening of pre-existing chronic conditions, insufficient or unnecessarily delayed prenatal care, returning to one’s country of origin for treatment of terminal illnesses, and inappropriate medication usages, such as inability to refill prescriptions or inappropriately using antibiotics obtained on the black market. Similar findings were reported by mixed methods studies of the public health effects of immigration policies on residents in North Carolina [15] and Arizona [28].

Additional barriers to health care women mentioned included inflexible work schedules, the lack of interpreters at some clinics and difficulties in finding someone to give them a ride or with navigating the transit system. Consequently, they treated health care as a last resort when their traditional remedies or over-the-counter treatment strategies failed them. The looming possibility of incurring financial debt produced stress, fear, and anxiety. Moreover, the inability to find reliable and affordable health care, especially for chronic conditions, caused women to experience depressive symptoms, which were compounded by the larger society’s narrative which treated them as outsiders and not a part of the community. They found solace in their personal networks, strengthened through their association with a church and its faith leaders. Despite the many obstacles in their personal lives, they expressed optimism for the future of their children, who were oftentimes fluent English speakers and US citizens by birth.

The study participants were immigrants who came to the US primarily as economic migrants. One of the limitations of this research is that we did not directly ask participants to report their immigration status, but fears of increased immigration enforcement were expressed by many participants. Questions about which type of identification they used for health care appointments were usually evaded, suggesting that asking about immigration status could lead to feelings of distrust. The participants included mostly women from Mexico (80%), with the others divided evenly between the countries of El Salvador, Guatemala, and Honduras. The women all had different reasons and rationale to explain their decision to come to the US, but this was beyond the scope of the interview questions about health care access. While participants acknowledged there were many problems with the health care system in the US in terms of affordable access for serious conditions, they expressed that there were even fewer specialty care services and lower quality of care in their home countries.

## Conclusion

This study allowed participants to share their experiences and challenges with accessing the US health care system. The Latina women discussed how their physical activity and eating patterns had changed for the worse since living in the US, but they retained many of the natural remedies they used to stay healthy and avoid getting sick. However, they would seek ‘black market’ sources for cheaper prescription drugs, like findings from a study on medication usage by immigrants in North Carolina [29]. Frequently cost was the driving factor affecting their health care decision making.

Since the interviews took place during the 2016 US Presidential campaign, the increased immigration enforcement activities by US Immigration and Customs Enforcement (ICE) had not yet occurred. Since the US presidential election, there have been many public meetings in different Latino communities in South Carolina to address people’s fears and to answer immigration law questions in response to the imminent threat posed by increased ICE activity. There is a need for more longitudinal studies to measure the direct and indirect effects of increased immigration enforcement activities on health care access for Latino immigrants. These types of studies should measure the long-term effects of immigration policies on health outcomes and employ mixed methods research designs. Moreover, in addition to the need for immigration policy reform, future US health care policy should identify solutions for the health care and health insurance needs of the 11 million undocumented people living in the US to improve health equity for all.

## Additional file

Additional file 1: Qualitative Interview Instrument. This instrument contains close-ended questions on health history and demographic variables and open-ended questions focusing on access to healthcare. (DOCX 33 kb)

## Abbreviation

ACA: The Patient Protection and Affordable Care Act
