# Supplementary material for: Access to health care for uninsured Latina immigrants in South Carolina
Source: BMC Health Serv Res. 2018 May 2;18:310. doi: 10.1186/s12913-018-3138-2 (PMC5930513; doi:10.1186/s12913-018-3138-2)
Supplement: Supplementary file 1 — Qualitative Interview Instrument. This instrument contains close-ended questions on health history and demographic variables and open-ended questions focusing on access to healthcare. (DOCX 33 kb) [file 12913_2018_3138_MOESM1_ESM.docx]

Place: _____________________­­­­­­­­­­­­­­­­­­­­­­­­­

Date: _____/_____/______

**The purpose of this interview is to evaluate access to healthcare for Latina women as well as their use of alternative medicines and therapies. This interview is part of a research project at MUSC/Medical University of South Carolina that is led by Dr. John Luque. The interview takes about 30 minutes to complete and is voluntary and confidential. We will be recording part of the interview. No identifying information (name, address, or other) will be collected.**

**Thank you for agreeing to participate in the interview. Your responses will help our research team design innovative solutions to improve access to healthcare and prevent and manage diseases impacting so many Latina women.**

**Your participation in this research project is completely voluntary: you may stop at any time or choose not to answer any question. If you have any questions about this project, please contact Program Coordinator Carolina Davila, 843-513-4483 or Dr. Luque, 843-876-2248.**

**A. YOUR HEALTH CARE**

A1: Is there a particular doctor, nurse, or other health professional that you see most often?

🞏 Yes

🞏 No

A2. Do you have any kind of health care coverage, including health insurance, prepaid plans such as HMOs, or government plans such as Medicare?

🞏 Yes

🞏 No

A3. Since October of 2013, have you tried to get health insurance for yourself through the new federal health care law (e.g., from healthcare.gov or a state website)?

🞏 Yes

🞏 No

🞏 I don’t know

A4. A routine checkup is a general physical exam, not an exam for a specific injury, illness, or condition. About how long has it been since you last visited a doctor for a routine checkup?

🞏 Within past year

(anytime less than 12 months ago)

🞏 Within past 2 years

(1 year but less than 2 years ago)

🞏 Within past 5 years

(2 years but less than 5 years ago)

🞏 5 or more years ago

🞏 Don’t know

🞏 Never

**B: YOUR OVERALL HEALTH**

B1. In general would you say your health is …

🞏 Excellent

🞏 Very Good

🞏 Good

🞏 Fair

🞏 Poor

B2. Overall, how confident are you about your ability to take good care of your health?

🞏 Completely confident

🞏 Very confident

🞏 Somewhat confident

🞏 A little confident

🞏 Not confident at all

B3. In the past 12 months, not counting times you went to an emergency room, how many times did you go to a doctor, nurse, or other health professional to get care for yourself?

🞏 None

🞏 1 time

🞏 2 times

🞏 3 times

🞏 4 times

🞏 5-9 times

🞏 10 or more times

B4. Has a doctor or other health professional ever told you that you had any of the following medical conditions:

|  | Yes | No |
| --- | --- | --- |
|  | ⮛ | ⮛ |
| a. Diabetes or high blood sugar?.… | 🞏 | 🞏 |
| b. High blood pressure or hypertension?……………………. | 🞏 | 🞏 |
| c. A heart condition such as heart attack, angina, or congestive heart failure?................................. | 🞏 | 🞏 |
| d. Chronic lung disease, asthma, emphysema, or chronic bronchitis?.................................... | 🞏 | 🞏 |
| e. Arthritis or rheumatism?................ | 🞏 | 🞏 |
| f. Depression or anxiety disorder?.... | 🞏 | 🞏 |
| g. Cancer | 🞏 | 🞏 |

B4. Do you have a family history (mother, father, sister, brother) of any of the following medical conditions:

|  | Yes | No |
| --- | --- | --- |
|  | ⮛ | ⮛ |
| a. Diabetes or high blood sugar?.… | 🞏 | 🞏 |
| b. High blood pressure or hypertension?……………………. | 🞏 | 🞏 |
| c. A heart condition such as heart attack, angina, or congestive heart failure?................................. | 🞏 | 🞏 |
| d. Chronic lung disease, asthma, emphysema, or chronic bronchitis?.................................... | 🞏 | 🞏 |
| e. Arthritis or rheumatism?................ | 🞏 | 🞏 |
| f. Depression or anxiety disorder?.... | 🞏 | 🞏 |
| g. Cancer | 🞏 | 🞏 |

**C: TOBACCO PRODUCTS**

C1. Does anyone in your household currently smoke cigarettes?

🞏 Yes

🞏 No

C2. Have you smoked at least 100 cigarettes in your entire life?

🞏 Yes

🞏 No 🡪 GO TO C6 below

C3. How often do you now smoke cigarettes?

🞏 Every day

🞏 Some days

🞏 Not at all 🡪 GO TO C6 below

C4. At any time in the past year, have you stopped smoking for one day or longer because you were trying to quit?

🞏 Yes

🞏 No

C5. Are you seriously considering quitting smoking in the next six months?

🞏 Yes

🞏 No

C6. Have you heard of any tests to find lung cancer before the cancer creates noticeable problems?

🞏 Yes

🞏 No

**D: WOMEN AND CANCER**

D1. A Pap test is an exam of your cervix (which is part of your uterus) to look for cervical cancer. Do you know how often you should get your Pap test?

🞏 Every year

🞏 Every two years

🞏 Every three years

🞏 Every four years

🞏 Every five years

D2. How long ago did you have your most recent Pap test to check for cervical cancer?

🞏 A year ago or less

🞏 More than 1, up to 2 years ago

🞏 More than 2, up to 3 years ago

🞏 More than 3, up to 5 years ago

🞏 More than 5 years ago

🞏 I have never had a Pap test

D3. A mammogram is an x-ray of each breast to look for breast cancer. When did you have your most recent mammogram to check for breast cancer, if ever?

🞏 A year ago or less

🞏 More than 1, up to 2 years ago

🞏 More than 2, up to 3 years ago

🞏 More than 3, up to 5 years ago

🞏 More than 5 years ago

🞏 I have never had a mammogram

D4. Has a doctor ever told you there are different tests, such as colonoscopy or blood stool tests to detect colorectal cancer?

🞏 Yes

🞏 No

🞏 I have never discussed these tests with a doctor

D5. How long ago did you have your most recent screening test to check for colorectal cancer?

🞏 A year ago or less

🞏 More than 1, up to 2 years ago

🞏 More than 2, up to 3 years ago

🞏 More than 3, up to 5 years ago

🞏 More than 5 years ago

🞏 I’ve never had a colorectal cancer screening test

D6. What type of colorectal cancer screening test was it?

🞏 Colonoscopy

🞏 Blood stool test

D7. As far as you know, which of the following statements are true or false about medical tests or exams such as colonoscopies, mammograms, and Pap tests that check for early signs of cancer?

|  | True | False | Do not know |
| --- | --- | --- | --- |
|  | ⮛ | ⮛ | ⮛ |
| a. These tests can definitely tell that a person has cancer……. | 🞏 | 🞏 | 🞏 |
| b. When a test finds something abnormal, more tests are needed to know if it is cancer…………………………. | 🞏 | 🞏 | 🞏 |
| c. When a test finds something abnormal, it is very likely to be cancer…………………………. | 🞏 | 🞏 | 🞏 |

D8. When did you have your most recent blood glucose test to check for diabetes, if ever?

🞏 A year ago or less

🞏 More than 1, up to 2 years ago

🞏 More than 2, up to 3 years ago

🞏 More than 3, up to 5 years ago

🞏 More than 5 years ago

🞏 I have never had a blood glucose test

D9. Do you know your blood pressure? For example, normal is 120/80? ___/___

D10. Where do you get your blood pressure checked?

**E. QUALITATIVE INTERVIEW GUIDE**

***Note to Interviewer: Begin recording***

E1. What kind of things do Latino/a community members do to stay healthy?

*Probes*: Exercising, taking vitamins, etc.?

E2. What should we do to stay physically healthy?

*Probes*: Do you exercise and if so, how often?

E3. What should we do to stay mentally healthy?

E4. What should we do to stay spiritually healthy?

*Probe*: In what way does your religion help or hinder you in seeking healthcare?

E5. What are some of the reasons for not doing what we are supposed to be doing to be healthy?

E6. How do we know when we are sick?

E7. Do you have control over your own health?

*Probe*: What are the biggest worries that Latinos/as have about their health?

E8. What are the top three health problems faced by adult Latinos/as in the U.S.?

E9. What kinds of things do doctors tell us to do to be healthy?

E10. What kinds of things do our mothers and grandmothers tell us to do to be healthy?

E11. What are some kinds of natural remedies we use to stay healthy?

E12. Where do you go to find the medicines you need to stay healthy?

E13. Where do you go for healthcare?

*Probes*: Do you see other types of healthcare providers besides doctors/nurses?

Have you used family planning? Have you used prenatal care?

E14. What challenges do you have to get doctor’s appointments, go to the doctor, and get the care that you need?

E15. If you have any health problems (for example, diabetes, high blood pressure, asthma), what makes it difficult to manage your illness?

E16. Tell me about the most recent visit to a healthcare professional (doctor, nurse) in the U.S..

*Probe*: When was it? What was the reason? Was your health problem resolved?

E17. What do you think about the costs of healthcare in the U.S.?

*Probes*: How does the cost affect use of emergency services? Routine healthcare?

E18. What are some of the reasons you go to seek healthcare?

E19. What makes you seek help when you are sick?

E20. How good a job do healthcare facilities do with providing Spanish-language services?

*Probe*: Spanish language forms? Educational brochures in Spanish?

E21. Have you felt discriminated about your Hispanic Ethnicity?

*Probes*: In healthcare centers? At work? In stores?

E22. Do you feel anxious? Where do you feel the safest?

E23. Is there anything else about access to healthcare in the U.S. that you want to say?

***Note to Interviewer: Stop recording***

**F: YOU AND YOUR HOUSEHOLD**

F1. What is your age?

______ Years old

F2. What is your occupational status?

Mark 🗷 only one.

🞏 Employed

🞏 Unemployed

🞏 Homemaker

🞏 Student

🞏 Retired

🞏 Disabled

🞏 Other-Specify

F3. What is your marital status?

Mark 🗷 only one.

🞏 Married

🞏 Living as married

🞏 Divorced

🞏 Widowed

🞏 Separated

🞏 Single, never been married

F4. What is the highest grade or level of schooling you completed?

🞏 Less than 8 years

🞏 8 through 11 years

🞏 12 years or completed high school

🞏 Post high school training other than college (vocational or technical)

🞏 Some college

🞏 College graduate

🞏 Postgraduate

F5. What city and country were you born in?

City ________________

Country _____________

F6. What year did you come to live in the United States?

____________ Year

F7. How well do you speak English?

🞏 Very well

🞏 Well

🞏 Not well

🞏 Not at all

F8. How well do you speak Spanish?

🞏 Very well

🞏 Well

🞏 Not well

🞏 Not at all

F9. Do you speak any other languages?

🞏 Yes __________ (specify)

🞏 No

F10. What language do you prefer to speak?

🞏 English

🞏 Spanish

🞏 Both equally

F11. Which language do you speak the most at home?

🞏 English

🞏 Spanish

🞏 Both equally

F12. What was your first language as a child?

🞏 English

🞏 Spanish

🞏 Other (specify) ______

F13. Many people have difficulty reading in either English or Spanish.

Do you read any English?

🞏 Yes, anything

🞏 Some

🞏 Very little

🞏 None

F14. How would you rate your ability to read in Spanish?

🞏 Very good

🞏 Good

🞏 Ok

🞏 Poor

🞏 Very poor

F15. Including yourself, how many people live in your household?

______ Number of people

F16. How many children under the age of 18 live in your household?

______ Number of children under 18

F17. Do you currently rent or own your home?

🞏 Own

🞏 Rent

🞏 Occupied without paying rent

F18. Does anyone in your family have a working cell phone?

🞏 Yes

🞏 No

F19. Does anyone in your family have; check all that apply.

🞏 Smartphone

🞏 Tablet

🞏 Computer

🞏 Email

🞏 I do not have any of these

F20. Thinking about members of your family living in this household, what is your combined annual income, meaning the total pre-tax income from all sources earned in the past year?

🞏 $0 to $9,999

🞏 $10,000 to $14,999

🞏 $15,000 to $19,999

🞏 $20,000 to $34,999

🞏 $35,000 to $49,999

🞏 $50,000 to $74,999

🞏 $75,000 to $99,999

🞏 $100,000 to $199,999

🞏 $200,000 or more

🞏 I don’t know

F21. What is your zipcode? ___________
